# Potato virus Y; the Andean connection

**DOI:** 10.1093/ve/vez037

**Published:** 2019-09-23

**Authors:** Segundo Fuentes, Roger A C Jones, Hiroki Matsuoka, Kazusato Ohshima, Jan Kreuze, Adrian J Gibbs

**Affiliations:** 1 Crop and System Sciences Division, International Potato Center (CIP), Apartado 1558, Lima 12, Peru; 2 Institute of Agriculture, University of Western Australia, 35 Stirling Highway, Crawley, WA; 3 Department of Primary Industries and Regional Development, 3 Baron-Hay Court, South Perth, WA, Australia; 4 Laboratory of Plant Virology, Department of Applied Biological Sciences, Faculty of Agriculture, Saga University, 1-banchi, Honjo-machi, Saga, Japan; 5 Emeritus Faculty, Australian National University, ACT2601, Australia

**Keywords:** potato virus Y, phylogenetics, dating, history, Colombian exchange

## Abstract

*Potato virus Y* (PVY) causes disease in potatoes and other solanaceous crops. The appearance of its necrogenic strains in the 1980s made it the most economically important virus of potatoes. We report the isolation and genomic sequences of 32 Peruvian isolates of PVY which, together with 428 published PVY genomic sequences, gave an alignment of 460 sequences. Of these 190 (41%) were non-recombinant, and 162 of these provided a dated phylogeny, that corresponds well with the likely history of PVY, and show that PVY originated in South America which is where potatoes were first domesticated. The most basal divergences of the PVY population produced the N and C: O phylogroups; the origin of the N phylogroup is clearly Andean, but that of the O and C phylogroups is unknown, although they may have been first to establish in European crops. The current PVY population originated around 156 CE. PVY was probably first taken from South America to Europe in the 16th century in tubers. Most of the present PVY diversity emerged in the second half of the 19th century, after the *Phytophthora infestans* epidemics of the mid-19th century destroyed the European crop and stimulated potato breeding. Imported breeding lines were shared, and there was no quarantine. The early O population was joined later by N phylogroup isolates and their recombinants generated the R1 and R2 populations of damaging necrogenic strains. Our dating study has confirmed that human activity has dominated the phylodynamics of PVY for the last two millennia.

## 1. Introduction

The potato is now one of the most important food crops globally (Devaux, Kromann, and Ortiz 2014), and virus diseases are a major factor constraining its production, especially in developing countries (Jones 2014). *Potato virus Y* (PVY; genus *Potyvirus*) causes disease in potato and a wide range of other solanaceous species, including the important crop species tobacco, tomato, and pepper (Kerlan 2006). PVY became the most economically important virus of potatoes in the 1980s after PVY recombinants that cause ‘potato tuber necrotic ringspot disease’ (PTNRD; Kus 1992) appeared and proved difficult to control in seed potato crops. PVY is carried in infected tubers, is transmitted locally non-persistently by aphids and is occasionally transmitted by plant-to-plant contact. In addition to diminishing tuber quality through PTNRD, it decreases tuber yields by up to 80 per cent (Loebenstein et al. 2001; Stevenson et al. 2001; Kerlan 2006; Kerlan and Moury 2008; Ogawa et al. 2008, 2012; Gray et al. 2010; Karasev and Gray 2013; Jones 2014; Coutts and Jones 2015).

Several biological strain groups (i.e. pathotypes) and phylogroups of PVY have been distinguished using biological and gene sequence differences, respectively. Biological strain groups from potato are recognized firstly by the necrotic phenotypes that develop when inoculated to potato cultivar ‘differentials’ carrying strain-specific hypersensitivity genes, and then by whether they cause systemic veinal necrosis symptoms in tobacco. Thus, PVY^C^, PVY^O^, PVY^Z^, and PVY^D^ cause necrotic phenotypes with strain-specific hypersensitivity genes *Nc, Ny*, *Nz*, or putative *Nd*, respectively. PVY^N^ and PVY^E^ overcome *Nc, Ny*, and *Nz* (yet to be tested with putative *Nd*), but are separated by the symptoms they cause in infected tobacco plants, only PVY^N^ inducing veinal necrosis in this species (Jones 1990; Singh et al. 2008; Karasev and Gray 2013; Gray and Power 2018; Jones and Vincent 2018).

Gibbs et al. (2017) found that all PVY isolates for which complete genome sequences were then known fall into five distinct phylogroups (N, C, O, R1, and R2). Half of all the isolates form the N, C, and O phylogroups, most isolates of which are non-recombinant, whereas the R1 and R2 phylogroups are all recombinants with two underlying patterns of recombination; both have genomes with a 5′-terminal region from a N phylogroup parent (1–2,604 of the 9,201 nucleotides, nts) and an O group core region (2,605–5,505 nts), but whereas the R1 isolates have a 3′-terminal region (5,506–9,201 nts) from the O phylogroup parent, those of the R2 isolates are from an N parent. The R1 and R2 isolates are of considerable economic importance as most, but not all, cause PTNRD, although not all PTNRD is caused by R1 and R2 isolates. Thus the two nomenclatural systems, host genetics versus virus genetics, mostly coincide, but not always. For example, PVY^D^ belongs to phylogroup C, non-recombinant PVY^z^ isolates to phylogroup O (Jones and Kehoe 2016; Kehoe and Jones 2016), and recombinant PVY^z^ isolates to R2 (Kerlan et al. 2011; Karasev and Gray 2013). Furthermore, as described below, the seven isolate cluster we call O3 contains two C pathotype isolates (KP691321 and KP691324; Kehoe and Jones 2016), one D pathotype isolate (KP691329; Kehoe and Jones 2016) and Salazar, Bartolini, and Flores (2000) reported that two Peruvian isolates collected in 1996 were of PVY^NTN^ yet the P1 gene sequence of one of them showed that it was from the O phylogroup.

The PVY sequences reported to GenBank come from all continents except Antarctica. However, in January 2018, none had been reported from the Andean region of Bolivia and Peru, which is where potatoes were originally domesticated, and where the greatest genetic diversity of cultivated and wild potato species occurs (Hawkes1978, 1990; Brown 1993; Ovchinnikova et al. 2011; Brown and Henfling 2014). *Solanum tuberosum* is the only potato species widely cultivated outside this region whereas additional cultivated potato species and a large number of land races are widely grown there. Although they were originally grouped as nine species, they were recently rearranged into four (*S.* *tuberosum*, *Solanum* *ajanhuiri*, *Solanum* *curtilobum*, and *Solanum* *juzepczukii*) (Ovchinnikova et al. 2011). *Solanum* *tuberosum* has a haploid chromosome number of twelve, and ploidy levels ranging from diploid to hexaploid, and this was formerly the main property used to subdivide potatoes belonging to the recently enlarged species, *S.* *tuberosum*, into five species (Hawkes 1978, 1990; Brown 1993). As the greatest diversity of cultivated and wild potato species occurs in the Andean region, it is important to know whether PVY is similarly diverse in the same region as this could imply that they had co-evolved there. Moreover, this has already been reported to be so for two other common potato viruses, *Potato virus S* (PVS; genus *Carlavirus*) and *Potato virus X* (PVX, genus *Potexvirus)* (Kutnjak et al. 2014; Santillan et al. 2018). In the Andean region, PVY infection is common in potato plantings at altitudes below 3,000 masl (meters above sea level) but not above. This apparently reflects the greater abundance of its aphid vectors below this level, whereas the colder higher altitudes do not limit spread of contact-transmitted viruses PVS and PVX.

An understanding of the factors that produced the demography of the present PVY population requires knowledge of the history of both the virus and its hosts. There is a well recorded history of the potato and its wild relatives (Hawkes 1978, 1990; Brown 1993; Hawkes and Fransisco-Ortega 1993), but a better understanding of the virus and its interactions with its hosts requires a dated phylogeny of the virus too. That by Visser, Bellstedt, and Pirie (2012) concluded that the ‘time to most recent common ancestor’ (TMRCA) of the extant population was around the time when potatoes were first introduced from South America to Europe (i.e. the 16th century). A similar study with more sequences by Gibbs et al. (2017) inferred that the TMRCA of the virus was earlier, around 1,000 CE. However, the confidence limits of both estimates were large and overlapping. Gibbs et al. (2017) concluded that isolates obtained from the Andean center of potato domestication would probably be more temporally informative and therefore essential for successful dating.

In this paper, we report thirty-two additional isolates of PVY from the Peruvian region of the Andes, and the genomic sequences obtained from them. These, together with a further eight from the Colombian region published recently in GenBank, have allowed much more informative dating analyses to be done. The analyses indicate that the TMRCA of the world PVY population is earlier than previously proposed, and we interpret the historical events that the virus and its hosts have possibly shared since then.

## 2. Materials and methods

### 2.1 Sample collection, RNA library preparation, sequencing, and reverse transcription-polymerase chain reaction (RT-PCR) testing

Leaf samples were collected from potato plants showing foliage symptoms indicative of virus infection. Each sample was placed in a separate labeled paper filter bag, nine of which were placed together in a zip-lock plastic bag filled with 100 g of dehydrated silica-gel for rapid desiccation. The silica-gel was changed after 24–48 h and the combined samples were taken to CIP in Lima for processing.

Total RNA was extracted from each sample using Trizol as instructed by the manufacturer. The large RNA fraction was precipitated by adding 1 volume of 4 M LiCl4 at ∼4 °C (on ice) overnight, followed by centrifugation. The remaining small RNA fraction was subsequently precipitated by adding 1 volume of isopropanol followed by centrifugation. Small RNAs were separated on 3.5 per cent agarose gels and bands corresponding to ∼20–25 nts excised and purified using quantum prep freeze and squeeze columns (Bio-Rad). Small RNA libraries were prepared using the protocol of Chen et al. (2012) and sent for sequencing on a HiSeq4000 by a commercial provider (Fasteris Life Sciences SA, Plan-les-Ouates, Switzerland). Small RNA sequences were analyzed using VirusDetect (Zheng et al. 2017) v1.6 to identify all viruses infecting the plants, and samples in which PVY was identified were selected for further analysis. We specifically selected samples that showed no signs of possible mixed infections with other isolates and each was double checked by re-aligning of sRNA reads to the consensus using Geneious software, in all cases, and the majority consensus sequence was saved. It has previously been shown that sRNA sequences truthfully represent the viral sequence and its variants in PVY (Kutnjak et al. 2015). The large RNA fraction was used to confirm PVY presence using CIP PVY primers PVY-CP_F (5′-CAACTGTGATGAATGGGCTTAT-3′) and PVY-CP_R (5′-TCTCTGTGTTTTCCTCTTGTGT-3′), using standard procedures with an annealing temperature of 52.5 °C.

### 2.2 Sequence analysis

New and downloaded sequences were edited using BioEdit (Hall 1999) to extract the main open reading frames (ORFs). These were aligned, using the encoded amino acids as guide, by the TranslatorX online server (Abascal, Zardoya, and Telford 2010; http://translatorx.co.u;. 25 June 2019) with its MAFFT option (Katoh and Standley 2013). To search for non-PVY sequences with which to root phylogenies, the BLASTn and BLASTp online facilities of GenBank (Altschul et al. 1990) were used with sequences representing the N and C phylogroups, which are the most distantly related PVYs. Sequences were tested for recombinants using the full suite of options in Recombination Detection Program 4 (RDP4) with default parameters (Maynard Smith 1992; Holmes, Worobey, and Rambaut 1999; Padidam, Sawyer, and Fauquet 1999; Gibbs, Armstrong, and Gibbs 2000; Martin and Rybicki 2000; McGuire and Wright 2000; Posada and Crandall 2001; Martin et al. 2005, 2015; Boni, Posada, and Feldman 2007; Lemey et al. 2009); anomalies found by four or fewer methods and with greater than 10^−5^ random probability were ignored.

As recombinants are only generated when a host plant is simultaneously infected with both ‘parents’, recombinants may give useful information of when and where particular isolates or lineages were geographically co-located, and the most recently divergent of the two ‘parental’ lineages gives the maximum date of the recombinant event. These events and dates were obtained by separating the recombinant-containing alignment into two parts; one part containing the sequence of only one parent, and the other part containing the sequence of only the other parent. Trees calculated from these subalignments were then compared using Figtree (http://tree.bio.ed.ac.uk/software/figtree/; 12 May 2018) and the relative positions (i.e. relative dates) of individual nodes compared using PATRISTIC (Fourment and Gibbs 2006).

The best-fit substitution models for PVY sequences were assessed using Mega 7 (Kumar, Stecher, and Tamura 2016), and found to be GTR + Г_4_ + I (Tavaré 1986) for nucleotide sequences and LG + Г_4_ + I (Le and Gascuel 2008) for their encoded amino acid sequences. Phylogenetic trees were inferred using the neighbor-joining (NJ) option in ClustalX (Jeanmougin et al. 1998), the SplitsTree method (Huson and Bryant 2006), and PhyML 3.0 (ML) (Guindon and Gascuel 2003). In PhyML, the statistical support for their topologies was assessed using the method of Shimodaira and Hasegawa (1999). Most isolate collection dates (Supplementary Material) were obtained from GenBank files or from personal communications (Gibbs et al. 2017). The presence of a linear temporal signal was checked using the TempEst program (Rambaut et al. 2016), and estimates of the date of the TMRCA obtained by the ‘Least Squares Dating’ (LSD) method of To et al. (2016) using Version lsd-0.3beta. The presence of a non-linear temporal signal was checked, and the TMRCA estimated, using the probabilistic methods of BEAST v1.8.2 and Tracer v 1.6 (Drummond et al. 2012); for checking the signal, ten independently date-randomized replicates were obtained in BEAST analyses (Ramsden, Holmes, and Charleston 2008; Duchêne et al. 2015). Bayes factors (BFs) were used to select the best-fitting molecular-clock model and coalescence priors for the tree topology and node times. We compared strict and relaxed (uncorrelated exponential and uncorrelated lognormal) molecular clocks, as well as four demographic models (constant population size, expansion growth, exponential growth, and the Bayesian skyline plot). Posterior distributions of parameters, including the tree, were estimated from Markov Chain Monte Carlo (MCMC) samples taken every 10^4^ iterations from a total of 10^8^ iterations after discarding the first 10 per cent, and checked using Tracer v. 1.6 (http://tree.bio.ed.ac.uk/software/tracer/; 27 June 2019). The 'maximum clade credibility tree’ provided the TMRCA. Trees were drawn using Figtree Version 1.3 (http://tree.bio.ed.ac.uk/software/figtree/; 12 May 2018) and a commercial graphics package, and pairs of trees were compared using PATRISTIC (Fourment and Gibbs 2006) to test for mutational saturation and confirmed by the method of Xia (2013). The dates of nodes in trees were compared using the ratios of the mean pairwise patristic distances (Fourment and Gibbs 2006) of all sequences (i.e. tips) connected through individual nodes; the date of one node providing estimates of others in the same tree.

## 3. Results

### 3.1 Peruvian isolates

**Figure 1.**
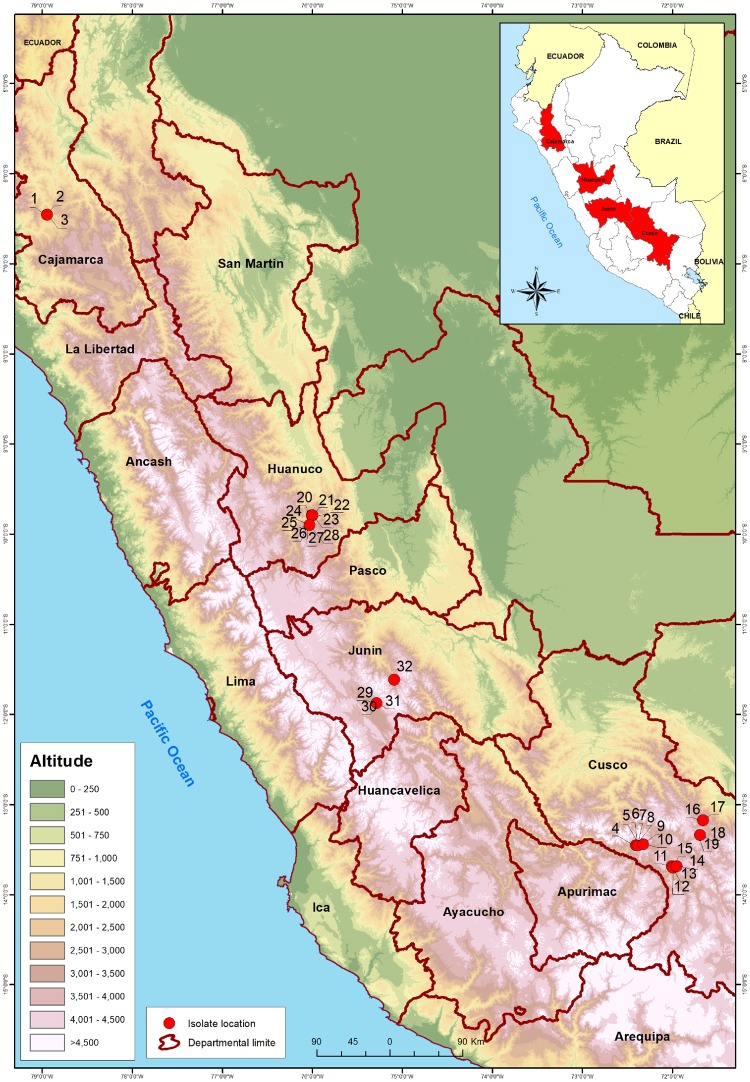
Map of potato sample collection sites in the Andean Highlands of Peru showing where PVY was detected (red spots). The numbers clustered around each collection site in this Figure indicate where each individual infected sample came and correspond to those in Table 1. The names marked on the map are those of the countries regional departments (red lines are departmental boundaries). Inset shows an outline map of the entire country and neighboring regions of the five countries with land borders.

In 2016, leaf samples were collected from 552 potato plants showing symptoms of mosaic, leaf deformation and/or stunting growing in fields at altitudes of 2,443–3,916 masl in four departments in the northern (Cajamarca), central (Huanuco, Junin), and southern (Cusco) Andean highlands of Peru (Fig. 1). The plants sampled included eight potato cultivars bred at CIP or at Peruvian national institutions from *S. tuberosum* ssp. *tuberosum* (*S.t.t.)* × *andigena* (*S.t.a.*) crosses and five native potato land races (belonging to *S.t.a., Solanum* *phureja, Solanum* *stenotomum* ssp. *goniocalyx, S. × chaucha* using the former species nomenclature). Viruses infecting them were identified by small RNA sequencing and their ‘viromes’ (http://potpathodiv.org/index.html; 27 June 2019) assembled. A number of viruses, with customary acronyms PVX, PVY, PVS, PVB, PVV, APMoV, PLRV, PVA, PMTV, PYV, APLV, and APMMV (listed in order of frequency), were identified in the study, but this paper will consider only one PVY. It was the second most common virus after PVX and detected in 235 (∼43%) of the samples, but many of these could not be resolved into individual or high quality complete sequences, due to mixed infection with multiple genotypes or gaps in the genome coverage by the RNA data respectively. Thirty-two samples were selected for analysis in this study as they yielded (near) complete genomic sequences and were only infected by a single PVY genotype, which was confirmed to be present in them by RT-PCR testing (Table 1). The numbers of sites (fields) providing the infected samples were one in the Cajamarca department (samples 1–3), two each in the Huanuco (samples 20–28) and Junin (samples 29–32) departments, and six in the Cusco department (samples 4–19) (Table 1, Fig. 1). There were 28 samples from potato cultivars (numbers of samples in parentheses) Canchan (5), Capiro (1), Cica (10), Perricholi (6), and Yungay (6), all of which were *S.t.t.* *×* *S.t.a.* crosses. These cultivars were sampled in the following departments: Cajamarca (Perricholi), Huanuco (Canchan, Capiro, Yungay) Junin (Perricholi, Yungay), and Cusco (Canchan, Cica). There were also four samples of the native potato land race *S.t.a. cv* Ccompis, all from the Anta province in the Cusco department.

**Table 1.** Provenances of new *Potato virus Y* isolates used in this study.

Sample	Isolate	Host species	Cultivar/breeding line name	Peruvian region where sample was collected/obtained	Latitude	Longitude	Collection year	GenBank code	Phylogroup
1	Cca01	*S. tuberosum* ssp*. tuberosum × S. tuberosum* ssp*. andigena*	Perricholi	Chente, Huambos, Chota, Cajamarca	−6.45626	−78.94315	2016	MH795841	O3
2	Cca07	*S. tuberosum* ssp*. tuberosum × S. tuberosum* ssp*. andigena*	Perricholi	Chente, Huambos, Chota, Cajamarca	−6.45626	−78.94315	2016	MH795842	O3
3	Cca10	*S. tuberosum* ssp*. tuberosum × S. tuberosum* ssp*. andigena*	Perricholi	Chente, Huambos, Chota, Cajamarca	−6.45626	−78.94315	2016	MH795843	O3
4	Czo18	*S. tuberosum* ssp*. tuberosum × S. tuberosum spp. andigena*	Cica	Cartonpata, Limatambo, Anta, Cusco	−13.45326	−72.40752	2016	MH795844	N3
5	Czo24	*S. tuberosum* ssp*. andigena*	Ccompis	Ayaviri (Pampacona), Limatambo, Anta, Cusco	−13.45755	−72.39146	2016	MH795872	N1
6	Czo25	*S. tuberosum* ssp*. andigena*	Ccompis	Ayaviri (Pampacona), Limatambo, Anta, Cusco	−13.45755	−72.39146	2016	MH795871	N1xN2
7	Czo29	*S. tuberosum* ssp*. andigena*	Ccompis	Ayaviri (Pampacona), Limatambo, Anta, Cusco	−13.45755	−72.39146	2016	MH795870	N1
8	Czo31	*S. tuberosum* ssp*. andigena*	Ccompis	Ayaviri (Pampacona), Limatambo, Anta, Cusco	−13.45755	−72.39146	2016	MH795845	N1xN2
9	Czo37	*S. tuberosum* ssp*. tuberosum × S. tuberosum spp. andigena*	Cica	San Martin, Ancahuasi, Anta, Cusco	−13.44399	−72.32522	2016	MH795869	N1
10	Czo42	*S. tuberosum* ssp*. tuberosum × S. tuberosum spp. andigena*	Cica	San Martin, Ancahuasi, Anta, Cusco	−13.44399	−72.32522	2016	MH795846	N3
11	Czo46	*S. tuberosum* ssp*. tuberosum × S. tuberosum spp. andigena*	Cica	Tiwicte, Huanoquite, Paruro, Cusco	−13.68634	−72.0074	2016	MH795868	N1
12	Czo56	*S. tuberosum* ssp*. tuberosum × S. tuberosum spp. andigena*	Cica	Tiwicte 2, Huanoquite, Paruro, Cusco	−13.70214	−71.99798	2016	MH795867	N1
13	Czo64	*S. tuberosum* ssp*. tuberosum × S. tuberosum spp. andigena*	Cica	Tiwicte 2, Huanoquite, Paruro, Cusco	−13.70214	−71.99798	2016	MH795864	N1
14	Czo72	*S. tuberosum* ssp*. tuberosum × S. tuberosum spp. andigena*	Cica	Taray, Yaurisque, Paruro, Cusco	−13.68939	−71.95618	2016	MH795866	N1
15	Czo75	*S. tuberosum* ssp*. tuberosum × S. tuberosum spp. andigena*	Cica	Taray, Yaurisque, Paruro, Cusco	−13.68939	−71.95618	2016	MH795863	N3
16	Czo102	*S. tuberosum* ssp*. tuberosum × S. tuberosum* ssp*. andigena*	Canchan	Pilco, Challabamba, Paucartambo, Cusco	−13.17801	−71.66427	2016	MH795847	N3
17	Czo110	*S. tuberosum* ssp*. tuberosum × S. tuberosum* ssp*. andigena*	Canchan	Pilco, Challabamba, Paucartambo, Cusco	−13.17801	−71.66427	2016	MH795848	N3
18	Czo129	*S. tuberosum* ssp*. tuberosum × S. tuberosum spp. andigena*	Cica	Paucana, Colquepata, Paucartambo, Cusco	−13.34083	−71.69811	2016	MH795862	N3
19	Czo132	*S. tuberosum* ssp*. tuberosum × S. tuberosum spp. andigena*	Cica	Paucana, Colquepata, Paucartambo, Cusco	−13.34083	−71.69811	2016	MH795865	N1
20	Hco26	*S. tuberosum* ssp*. tuberosum × S. tuberosum spp. andigena*	Capiro	Acomayo road, Huanuco, Huanuco	−9.79563	−76.00653	2016	MH795849	O1
21	Hco28	*S. tuberosum* ssp*. tuberosum × S. tuberosum* ssp*. andigena*	Canchan	Pillao community, Minas Pillao, Huanuco, Huanuco	−9.79425	−75.99822	2016	MH795850	N2
22	Hco29	*S. tuberosum* ssp*. tuberosum × S. tuberosum* ssp*. andigena*	Canchan	Pillao community, Minas Pillao, Huanuco, Huanuco	−9.79425	−75.99822	2016	MH795851	O1
23	Hco30	*S. tuberosum* ssp*. tuberosum × S. tuberosum* ssp*. andigena*	Canchan	Pillao community, Minas Pillao, Huanuco, Huanuco	−9.79425	−75.99822	2016	MH795852	O1
24	Hco38	*S. tuberosum* ssp*. tuberosum × S. tuberosum* ssp*. andigena*	Yungay	Jircahuasi, Umari, Pachitea, Huanuco	−9.89973	−76.03152	2016	MH795853	O1
25	Hco39	*S. tuberosum* ssp*. tuberosum × S. tuberosum* ssp*. andigena*	Yungay	Jircahuasi, Umari, Pachitea, Huanuco	−9.89973	−76.03152	2016	MH795854	O1
26	Hco41	*S. tuberosum* ssp*. tuberosum × S. tuberosum* ssp*. andigena*	Yungay	Jircahuasi, Umari, Pachitea, Huanuco	−9.89973	−76.03152	2016	MH795855	O1
27	Hco42	*S. tuberosum* ssp*. tuberosum × S. tuberosum* ssp*. andigena*	Yungay	Jircahuasi, Umari, Pachitea, Huanuco	−9.89973	−76.03152	2016	MH795856	O1
28	Hco45	*S. tuberosum* ssp*. tuberosum × S. tuberosum* ssp*. andigena*	Yungay	Jircahuasi, Umari, Pachitea, Huanuco	−9.89973	−76.03152	2016	MH795857	N2
29	Jin94	*S. tuberosum* ssp*. tuberosum × S. tuberosum* ssp*. andigena*	Perricholi	Barrio Chilca, Santa Rosa de Ocopa, Concepcion, Junin	−11.87439	−75.28642	2016	MH795858	N2
30	Jin95	*S. tuberosum* ssp*. tuberosum × S. tuberosum* ssp*. andigena*	Perricholi	Barrio Chilca, Santa Rosa de Ocopa, Concepcion, Junin	−11.87439	−75.28642	2016	MH795859	N2
31	Jin96	*S. tuberosum* ssp*. tuberosum × S. tuberosum* ssp*. andigena*	Perricholi	Barrio Chilca, Santa Rosa de Ocopa, Concepcion, Junin	−11.87439	−75.28642	2016	MH795860	N2
32	Jin125	*S. tuberosum* ssp*. tuberosum × S. tuberosum* ssp*. andigena*	Yungay	Mariscal Castilla, Concepcion, Junin	−11.61828	−75.09151	2016	MH795861	N2

To assess the variation of PVY sequences found in the 203 remaining samples, we selected all samples yielding assembled contigs with a minimum of 850 nts covering the coat protein region and aligned them to representative sequences of phylogroups O1–3, N1–3, and R1 & R2. These corresponded to 121 samples, of which 65 included significantly differing contigs covering similar genomic regions, indicating mixed infections. Of those, 17 contained the 850 nts fragment used in our analysis and all represented combinations of isolates from the same phylogroups. Based on coat protein alignment 114 sequences belonged to N1 (from Cusco, Junin, and Cajamarca), 5 to N3 (from Cusco), and 1 each to O3 (Cajamarca) and O1/O2 (Cusco). Thus the thirty-two genome sequences utilized in this study represented well the variability found among the remaining PVY positive samples from the survey.

### 3.2 Phylogenetic analysis; identifying the dated non-recombinant (n-rec) sequences

**Figure 2.**
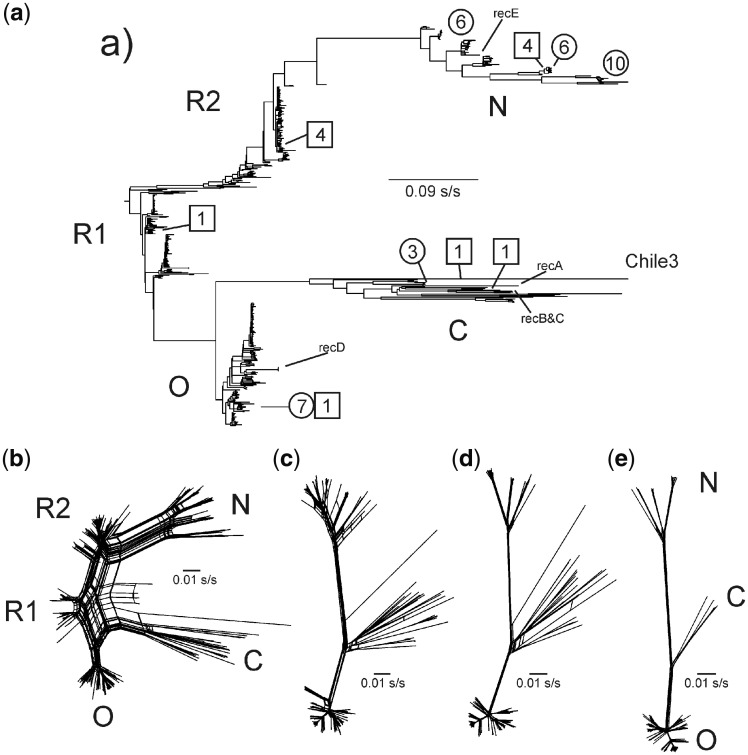
Various phylogenetic trees of PVY ORF sequences: (a) ML tree of 460 sequences; phylogroup clusters are marked, the numbers and positions of South American isolates are circled [this project] or boxed [from GenBank]; (b) SplitsTree phylogenies of the same 460 sequence alignment; (c) all 237 N, C, and O phylogroup sequences among the 460; (d) the 190 non-recombinant sequences among the 237; and (d) the 162 of the 190 ORFs that were used for dating analyses as their collection dates were known and they did not contribute large ‘residual’ variances to TempEst analyses.

**Figure 3.**
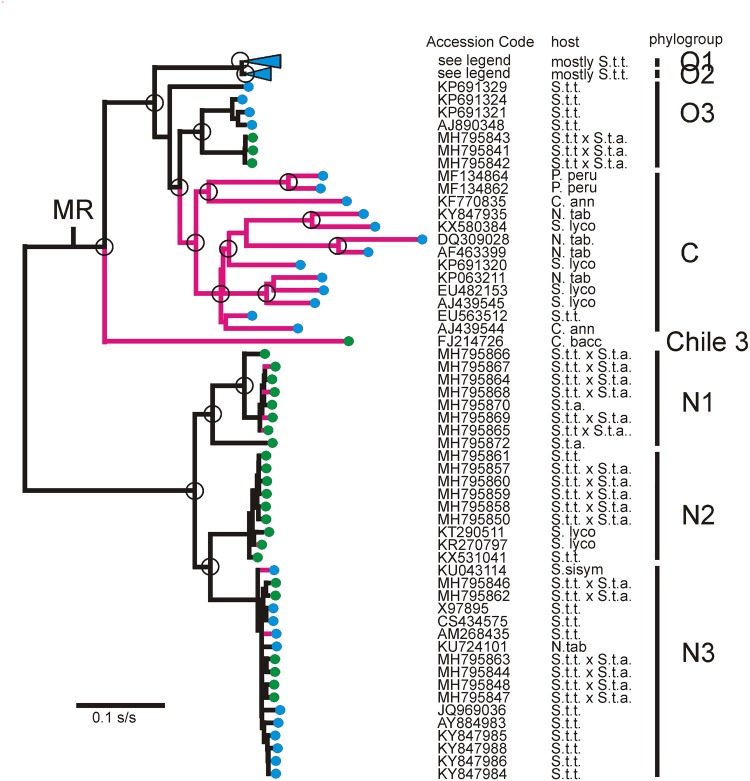
A phylogram illustrating the ML relationships of the 190 non-recombinant ORFs of the N, C, and O phylogroups shown in Fig. 2d. Most O isolate branches were collapsed to their two basal branches (blue triangles), the upper one (O1) represents 125 sequences and the lower (O2) 11 sequences; their Accession Codes are given in the Supplementary Material. The red branches show the isolates that contributed large ‘residual’ variances to TempEst analyses, and were removed to form the 162 dated ORFs dataset (Fig. 2e) for BEAST analysis. The tree was drawn with the midpoint root (MR) positioned so the major O and N phylogroup clusters fell on the same region of the *X*-axis (see text). Isolates collected in South America are marked with green disks, and those from elsewhere in the world are blue. The abbreviated host names: C. ann., *Capsicum annuum*; C. bacc., *Capsicum baccatum*; N. tab., *Nicotiana tabacum*; P. peru., *Physalis peruviana*; S. lyco., *Solanum lycopersicum*; S. sisym., *Solanum sisymbriifolium*; S.t.t., *Solanum tuberosum ssp. tuberosum*; S.t.a., *Solanum tuberosum ssp. andigena;* V. vin., *Vitis vinifera*.

The 32 new PVY sequences, all from Peru, together with 428 PVY sequences downloaded from GenBank in January 2018 gave a dataset of 460 genomic sequences (Supplementary Material). The principal ORFs from these genomic sequences produced an alignment 9,201 nts long. Fig. 2a shows the branch pattern of the maximum likelihood (ML) tree calculated from this alignment. It closely resembles in all major features the trees calculated earlier from smaller sets of PVY sequences published by Kehoe and Jones (2016; 73 sequences) and Gibbs et al. (2017; 240 sequences). The ML tree shows the same five major phylogroups, O, C, N, R1, and R2, reported by Gibbs et al. (2017). It included the thirty-two new Peruvian sequences (Fig. 2a; circled), twenty-two of which were from the N phylogroup and the other ten from the O phylogroup (Fig. 3), together with an additional eight sequences from Colombia, two from Brazil, and one each from Uruguay and Chile (all from GenBank and boxed in Fig. 2a).

The layered pattern of branches in the ML phylogeny (Fig. 2a) indicated that there were a large number of recombinants in the data, and this was confirmed by a SplitsTree analysis (Fig. 2b;Huson and Bryant 2006). The latter showed many parallel links within and between the major phylogroups; the NJ tree of the same data was closely similar in topology. When the 103 R1 and 120 R2 phylogroup sequences were removed, the remaining 237 O, C, and N phylogroup ORFs gave a much simpler SplitsTree diagram (Fig. 2c). These were then examined for additional phylogenetic anomalies using RDP4. Forty seven recombinant sequences were found, thirty-eight were intra-phylogroup recombinants, and only nine were inter-phylogroup recombinants. The remaining 190 non-recombinant (n-rec) sequences gave a much simpler SplitsTree diagram (Fig. 2d).

The ‘collection dates’ of 180 of the 190 n-rec N, C, and O phylogroup ORFs are known (Supplementary Material); they range from 1938 to 2016 CE (15% are pre-2000 CE). However, several of the dated sequences, mostly from non-potato hosts and in the C phylogroup, behaved anomalously in TempEst analyses (see below), and so were removed to give the 162 ORF alignment used for Bayesian dating analyses. Fig. 2e is the SplitsTree diagram of the 162 ORFs alignment.

### 3.3 Rooting the phylogeny of n-rec sequences

BLASTn and BLASTp searches using representative sequences from non-recombinant N and O phylogroup sequences found the most closely related genomic sequences to be those of pepper severe mosaic virus (PSMV) (AM181350; Ahn 2006) isolated from *Capsicum annuum* crops in the San Juan province of Argentina (Feldman and Gracia 1977), and then sunflower chlorotic mottle virus (JN863233) and bidens mosaic virus (KF649336); all 69–72 per cent identical in nucleotide and encoded amino acid sequences. The outgroup root for either the 190 n-rec ORFs or their encoded amino acid sequences were on the same ‘edge’ of their phylogenies, at the base of the N phylogroup and close to the divergence between the Chile 3 (FJ214726) sequence and the remainder of the C phylogroup. These root positions were also close to the midpoint roots calculated from ML trees of ORF sequences by Figtree and TempEst.

### 3.4 The phylogeny of the n-rec sequences

Figure 3 shows the ML tree of the 190 n-rec ORFs. In it, the major nodes with >0.9 SH support (Shimodaira and Hasegawa 1999) have been circled, but, for clarity, none of the distal nodes have been circled although most were also fully supported.

In all PVY phylogenetic trees, the N phylogroup is one of the basal sister lineages, and is the simplest of the two to interpret. The basal two-thirds of this lineage is represented by a single branch which dichotomized to form three major clusters of closely related sequences labeled N1, N2, and N3 in Fig. 3. Twenty three N isolates were South American, and only eleven from elsewhere. All except four came from *S.t.t.* or from potato hybrids; three of the exceptions were from other *Solanum* spp., and a fourth from tobacco. Divergence of the N lineage gave the N1 cluster, all the isolates of which came from Peru. Its sister lineage split into the N2 and N3 clusters. The N2 cluster contains isolates from Peru and Colombia, and only the N3 cluster includes any isolates from overseas, all of them closely related to others from South America. This clear pattern may be safely interpreted to indicate that the N phylogroup is widespread in the Andean region of South America, and that it has spread overseas only recently.

The other basal branch of the PVY phylogeny in Fig. 3 is more complex in that its first branch is to the Chile 3 ORF (FJ214726), and later it diverges to the O and C phylogroup clusters. The Chile 3 isolate is basal and is known from one complete sequence (FJ214726; Moury 2010) and partial sequences from three other isolates also from peppers in Chile (Sudarsono et al. 1993); parts of the genomes of these four isolates differ from one another by 1–6 per cent ID indicating that they represent a significantly variable population. All came from *Capsicum* *baccatum*; the Chile 3 isolate came from a pool of market fruits of unknown provenance (B. Moury, pers. comm.). The next most distal divergence produced the monophyletic and large O phylogroup; its O1 and O2 clusters are of 135 closely related isolates, and few from South America.

The interpretation of the other parts of the C and O lineage is less certain as it depends on whether one interprets the cluster of seven isolates called O3 in Fig. 3 as the sister group of the O1 and O2 clusters, or as the basal branches of the C phylogroup. We propose that the first possibility is correct because, in TempEst analyses described below, the O3 sequences had ‘residual variances’ similar to those of the N, O1, and O2 sequences (Supplementary Fig. S1) in correlations between tree positions and date of collection, whereas all C phylogroup isolates gave much larger residuals. Furthermore, the host from which each isolate was obtained gave similar groupings; the O1, O2, and O3 isolates were all from *S.t.t.* or potato hybrids except two from tobacco. In contrast, only one of thirteen hosts of the C phylogroup was *S.t.t.* Only eleven O phylogroup isolates were from South America (Supplementary Material), seven in the O1 cluster were from Peru, and formed a cluster with one from Brazil (JQ924285) and isolates from China, UK, and USA. All were distal to isolates not collected in South America suggesting that they might be ‘remigrants’; they could have escaped in new cultivars released in the 1960s and 1970s, before rigorous virus testing of all releases was instigated. The remaining three from Peru are in a sister clade to two from the UK and one from France within the O3 cluster, but with more basal branches to an isolate from ‘fingerling’ potato cv. Kipfler from Australia, and the O1 and O2 clade. Despite the small number of isolates involved this may indicate that the Peruvian O3s are remigrants; remigration is possible as over the last 70 years a large number of *S.t.t.* plants have been imported to Peru as tubers for breeding purposes, and would not have been tested rigorously for virus infection, before or after importation to Peru, until comprehensive regular virus testing was started in the 1980s, after *Potato spindle tuber viroid* was found in potato breeding lines from Peru in the late 1970s.

Since their appearance in Europe in the 1980s, the R1 and R2 phylogroup populations have become as widespread as those of the O phylogroup in most world regions although apparently not in the Andean region. As described above their ORF sequences have three distinct regions with different ancestry, and their core region (nts 2,605–5,502) is always closest to the same region of O phylogroup ORFs. Thus, this region is potentially able to provide comparative phylogenetic information for all PVY isolates, both n-rec and recombinant. However, when that region of all 460 ORFs (i.e. all phylogroups) was analyzed it was found to be much less phylogenetically informative than the 190 full-length ORFs as judged by SH values. Thus, it was not possible to unequivocally identify the earliest R1 or R2 sequences, or their dates, or their likely parents; as was similarly reported for a smaller number of sequences by Gibbs et al. (2017). Likewise their 5′ and 3′ terminal regions (nts 1–2,604 and 5,503–9,201) gave no unequivocal evidence about the identity of their parents or the first R1 and R2 isolates in either phylogenetic analyses or pairwise distance (SDT; Muhire, Varsani, and Martin 2014) estimates.

### 3.5 Dating the n-rec sequences

TempEst analyses of both NJ and ML trees of the 190 sequence dataset gave invalid TMRCA estimates; in the future, not in the past! Also, it was notable that all of the ORFs of the C phylogroup sequences, produced large residuals in the TempEst regression analyses (Supplementary Fig. S1). Indeed, given the well-established root of the phylogeny, this indicates that the C phylogroup ORFs had evolved around 2.5 times as fast as the N and O phylogroup ORFs (nucleotide diversity π  =  0.1124 for C phylogroup sequences compared with π  =  0.027 for O phylogroup sequences; Nei and Li 1979). This can be seen in Fig. 3, which was drawn with the midpoint root positioned so that the major O and N phylogroup clusters fell on the same region of the *X*-axis, thereby accentuating the difference in branch length (i.e. evolutionary rate) between the C phylogroup and the O and N phylogroups. We therefore tested the effect of sequentially removing the ORFs with the largest residuals, namely all the C phylogroup sequences and a few others, and obtained more sensible TMRCA estimates. Some of the 180 dated sequences lacked their full 5′ termini, so the shortest sequences were removed, and then the 5′ terminal 240 nts of all remaining ORFs were removed to decrease potential phylogenetic error arising from gaps. This resulted in an alignment of 162 almost-complete ORF sequences, 8,925 nts long, for dating analysis, and Fig. 2d confirmed that the SplitsTree branching pattern for this dataset was recombinant-free.

TempEst analyses of the 162 ORF dataset gave TMRCAs of 485 BCE for the NJ tree (correlation coefficient 0.139, *P* = 0.0394) and 1,278 BCE for the ML tree (correlation coefficient, 0.129, *P* = 0.0508). LSD analyses of the same data also gave sensible TMRCAs; NJ tree, 1344 BCE and ML tree 3383 BCE, but with large 95 per cent confidence intervals (CIs). These results indicated that, when the ORFs with noticeably faster evolutionary rates (i.e. C phylogroup) were removed, there was a negative linear correlation between collection date and phylogenetic position in both NJ and ML trees. Therefore, a Bayesian MCMC analysis of the 162 ORFs was done using the BEAST program.

Bayes factors were calculated for the dated, trimmed 162 ORFs, using twelve combinations of substitution models (http://beast.community/; 27 June 2019); three clock, and four population growth models (see Table 2). The results (Table 2) showed that the ‘relaxed uncorrelated lognormal’ clock with ‘expansion growth’ or ‘Bayesian skyline plot’ models gave adequate effective sample sizes with the largest Bayes factors calculated by either ‘path sampling’ or ‘stepping-stone’ sampling (Baele et al. 2012). The resulting TMRCA estimates are of 1841 and 1879 years before 2016; mean 1860 YBP or 156 CE. These TMRCAs were outside the range obtained from ten independently date-randomized replicates (Ramsden, Holmes, and Charleston 2008; Duchêne et al. 2015), confirming that the temporal signal in the data was adequate for BEAST dating. The TMRCA of 1860 YBP had 95 per cent confidence intervals of 1174.5–2640.5 YBP, which gives a ‘coefficient of variation’ (i.e. 95% CI range/TMRCA as %) of 78.8 per cent, whereas the earlier estimate reported by Gibbs et al. (2017) had a TMRCA of 3,603 (1,411–6,566) YBP, so was within the same range, but had a coefficient of variation of 151.4 per cent. The evolutionary rate of the C phylogroup could not be determined directly as too few dated sequences were available.

**Table 2.** Timescale analysis of ORF sequences using different BEAST programs.

Parameter				
Demographic model	Constant population size	Expansion growth	Exponential growth	Bayesian skyline plot
Path sampling (BF)	141.31	99.47	107.51	*179.87*
Stepping-stone sampling (BF)	152.94	*260.15*	110.3	185.96
TMRCA (95% CI)	1873 (1,090–2,608)	1841 (1,157–2,622)	1663 (1,071–2,384)	1879 (1,192–2,659)
TMRCA effective sample size	239	238	247	261
Substitution rate (nt/site/year)	9.66 × 10^−5^ (7.10 × 10^−5^–1.23 × 10^−4^)	9.30 × 10^−5^ (6.79 × 10^−5–^1.18 × 10^−4^)	9.89 × 10^−5^ (7.40 × 10^−5^–1.25 × 10^−4^)	9.16 × 10^−5^ (6.90 × 10^−5–^1.15 × 10^−4^)

Number of sequences: 162, sequence length: 8,913 nucleotides (nts), best-fit substitution model: GTR + I + Γ_4_, Best-fit clock model: relaxed uncorrelated lognormal. Best-fit population growth models were expansion growth and Bayesian skyline plot supported by the best Bayes factors (BFs) (italics) of path sampling and stepping-stone sampling, respectively (BEAST 1.8.2 and Tracer v1.6). The data sets passed date-randomization tests for temporal structure. Effective sample size of substitution rates was 128–167. TMRCA; years before 2016.

**Figure 4.**
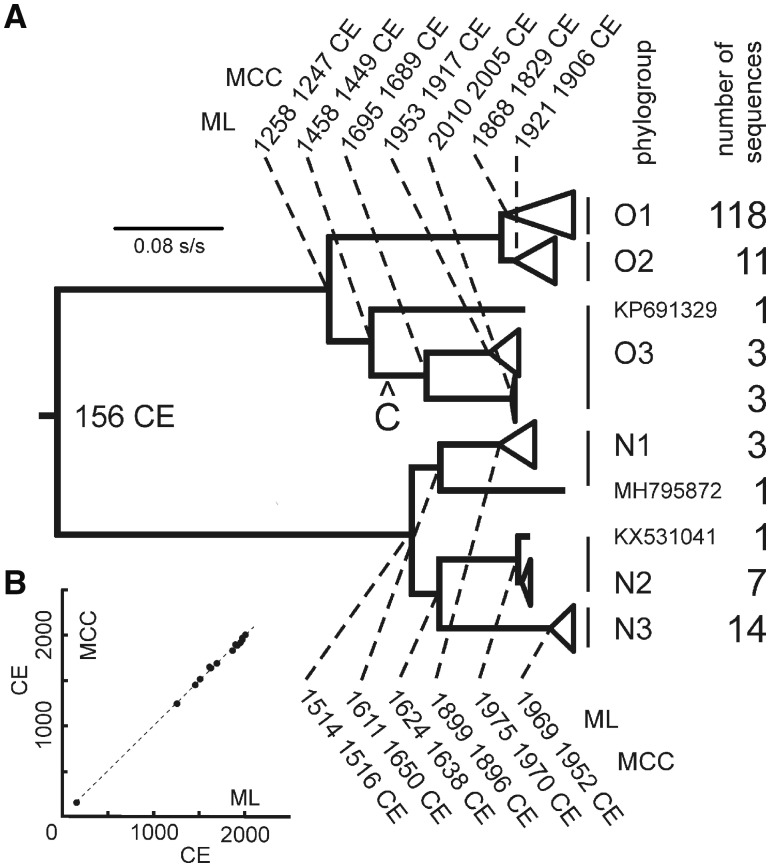
Dating. (A) A cartoon illustrating an ML phylogeny of the 162 sequences used for dating. The tree was dated using a BEAST estimated TMRCA of the basal node, 1860 YBP (Table 2). Most of the clusters of the 162 sequences are collapsed. Dates of major nodes with 0.99–1.00 SH statistical support were interpolated using the mean pairwise patristic distances of all sequences connected through each node together with the TMRCA of the basal node. The dates (CE, Common Era) from the maximum likelihood (ML) and maximum clade credibility (MCC) trees are given for each significantly supported node. The mean ‘coefficient of variation’ for individual estimates was around 9 per cent. The position of the node where the C phylogroup branch was attached is marked ‘C’. (B) A graph of the estimated ML dates plotted against the estimated MCC dates.

The mean TMRCA of PVY found by the Bayesian analyses was used as a datum to interpolate the dates of other nodes in the 162 ORF ML tree using patristic distances (Fourment and Gibbs 2006). Fig. 4 shows the nodes that were significantly supported (>0.99 SH), and their dates as calculated arithmetically from the TMRCA; relative dates were obtained from the ratios of the mean pairwise patristic distances of all sequences (i.e. tips) connected through individual nodes.

### 3.6 The recombinant sequences

About half the 460 PVY genomes are R1 and R2 recombinants with an O phylogroup core region (nts 2,605–5,505) and one or both terminal regions from an N phylogroup ‘parent’. The core region has few recombinants; one certain (KY848014) and three possible recombinants (AJ889868, AJ890343, and KY848014) among all 460. The core region, despite being almost one-third of the ORF in length, provided unreliable phylogenetic and dating information; well-supported clusters obtained with the complete ORFs overlapped when calculated from the core sequences as had been reported by Gibbs et al. (2017).

Recombinants may, however, also provide information about when and where their parental populations co-infected a host. Phylogenies calculated separately from the 5′ terminal (nts 1–2,604) and 3′ terminal (nts 5,506–9,201) regions of R1 and R2 phylogroup sequences both found that the N3, but not the N1 and N2, sequences were a sister cluster closest to the terminal regions of R1 and R2 sequences (SH support 1.0). N1 and N2 isolates have only been found in South America, whereas N3 isolates have also been found outside South America, and thus these relationships indicate that the recombination event(s) that produced the PTNRD-causing isolates of the R2 phylogroup likely occurred outside South America. However, Salazar (2006) suggests that isolates causing tuber necrosis may have ‘always been present in the Andes’ and ‘found in native potato cultivars in remote locations in the Andes that were never planted together or close to modern cultivars’, and ‘were probably spread with all the potato viruses when the crop was introduced to Europe in the XVI century’. Doubt remains as ‘The symptoms observed in the native cultivars under field conditions are not the same as those reported elsewhere. The symptoms in the Andean potatoes resemble more the russeting symptoms (reticulate surface cracking) that are observed with infections by some fungi and Streptomyces that are sometimes associated with mild to severe cracks on the tubers’.

Recombinants may also give an indication of relative node datings, and this is clearest when the parents are from different phylogroups, rather than when they are from the same phylogroup. Such information can be obtained for each recombinant by separating the alignment which contains it into two subalignments; one containing the parts of the alignment that include the ORF sequence of one ‘parent’ and the other containing the ORF sequence of the other ‘parent’. Trees calculated from the subalignments are then compared. The 237 N, C, and O phylogroup genomes included three CxO recombinants (n.b. major parent first), five OxN and one NxO, and, of course, no CxN recombinants, as expected as they mostly infected different host species.

The CxO recombinant AF237963 (recA in Fig. 2a) is the ‘pepper veinal necrosis’ strain-nnp found in Italy and isolated from *Capsicum* *annum* (Fanigliulo et al. 2005). In an ML phylogeny, its full ORF sequence forms a long basal branch among C phylogroup ORFs, but, in an RDP analysis, its 5′ terminal 375 nts region (4.1% of the ORF) was found by six methods to group with the homologous region of O phylogroup sequences with probabilities of random similarity of 10^−35^ to 10^−10^. Most of the ORF of AF237963 (nts 376–9,210) is most closely related to the homologous region of a C phylogroup isolate EU563512 collected from a *S. t.t* plant in the Netherlands in 1938, whereas the sequence of nts 1–375 is most closely related to that region of an O phylogroup isolate KY847936 collected from a *S. t.t* plant in the USA in 2005 (Green et al. 2017). Patristic distance comparisons using the dates in Fig. 4 indicate that the node linking the AF237963 and EU563512 lineages diverged about a century ago. The minor recombinant region grouped most closely with the isolate KY847936, whose ten closest relatives were also from *S. t.t* plants collected between 2000 and 2006 CE in the USA. Patristic distance comparisons, using 1868 CE as the date of the basal node of the O phylogroup (Fig. 4), indicated that the node linking the minor recombinant region and its nearest relatives formed in 1980.5 CE, namely just before AF237963 was collected in Italy. So, in summary, AF237963 most likely came from a doubly infected *S.t.t.* plant growing after 1981 in Europe or North America but not South America.

The CxO recombinant KR528584 (recB in Fig. 2a) is a PVY metagenome found in the publicly available transcriptome of a plant of *Vitis vinifera* clone ‘Tannat’ growing in Uruguay (Da Silva et al. 2013; Jo et al. 2015). The major ‘parental region’ of the ORF (nts 509–9,210) grouped closely with the C phylogroup genome EU482153, which was isolated from *Solanum* *lycopersicum* in Italy. Its minor O phylogroup region (nts 1–508) is most closely related to the homologous region of KP691319, which was collected from a *S.t.t.* plant in the UK in 1984. Patristic distance comparisons show that the node linking the minor recombinant region and its nearest relative was dated around 1945 CE. None of the nearest relatives of either the major or minor recombinant regions of KR528584 were collected in South America. Thus we conclude, that this recombinant, and the grapevine stock in which it was found, was a ‘remigrant’ recently imported into Uruguay from Europe, and not from the original Tannat stock carried to Uruguay from the Basque region by settlers in the 19th century (https://en.wikipedia.org/wiki/Tannat; 1 May 2019).

The third CxO recombinant KY848014 (recC in Fig. 2a) was isolated from a tobacco plant growing in the USA in 2011, and its nearest ‘parents’ show no links with South America. Its major and minor recombinant regions diverged from their nearest known relatives around 2008 CE and 1943 CE, respectively.

The six N and O phylogroup recombinants are actually records of only two isolates. One includes the PVY GenBank Reference Sequence NC_001616, which is an OxN recombinant (i.e. O major, N minor), and was derived, *in silico*, from three earlier GenBank submissions, A08776, D00441, and X12456. All four group on a single elongated branch in NJ and ML trees (recD in Fig. 2a). The virus was first isolated from *S.t.t.* in France in the 1980s by Robaglia et al. (1989). An identical sequence is recorded, probably incorrectly, as AF522296 from Egypt (Abdel El-Mohsen 2003). The minor recombinant region of this sequence, nts 7,722–8,150, is most closely related to the homologous region of AJ890346 from *S.t.t.* reported from Germany in 2003, and also four isolates (MH795846, MH795847, MH795848, and MH795863) of the N3 sublineage found in *S.t.t.* × *S.t.a.* crosses in Peru (i.e. this study). The major portion of the ORF is most closely related to KY848012 and other O phylogroup isolates collected in the USA, UK, and Australia from *S.t.t.* from 2003 to 2016. Patristic distance comparisons indicate that the node linking the major region of the recombinant’s ORF to its nearest relative formed in 1923 CE, but that joining the minor recombinant region to its relatives was no earlier than 1969. Thus, it is most likely that this recombinant was generated in Europe no earlier than 1969.

The sole NxO recombinant (AJ585197; recE in Fig. 2a), was isolated from *S.t.t*. in the UK and its genome sequence submitted to GenBank in 2003. Its major recombinant region is most closely related to X97895, which was isolated from *S.t.t*. growing in Switzerland (Jakab et al. 1997). ML trees of the separated regions confirmed that the closest isolate to its minor recombinant region is in the sequences AJ585195 and KP691326 which were both isolated from S.t.t. in the UK in 2003 and 1984, respectively. Its minor recombinant region is nts 7,748–8,150, which is almost the same region as in the reciprocal OxN recombinants discussed above. The possible reason for the unlikely occurrence of ‘reciprocal recombinants’ in a single region is that this region encodes two important and adjacent core motifs of the potyvirus RNA-dependent RNA polymerases; the -GNNSGQP- motif (Gibbs and Mackenzie 1997; Zheng et al. 2010) near its N-terminus and the universal -GDD- near its center.

## 4. Discussion

The sequences from Andean isolates of PVY that we report here, together with others from GenBank, nearly doubled the number of PVY genomic sequences available for analysis compared with a study only one year before (Gibbs et al. 2017). They increase greatly knowledge of PVY in South America.

PVY is the best studied member of the PVY lineage of potyviruses (Duarte et al. 2014), of which there are twenty-seven known members all except two of them isolated in the Americas, and seventeen of them having never been found anywhere else. They have a distinctive host range; most of the hosts are dicotyledonous asterids (Magallόn and Castillo 2009; Jiao et al. 2012; Zimmer and Wen 2013), none are rosids, and Solanaceae are not their only asterid hosts, as four are from Asteraceae. The PVY lineage arose in the Americas after diverging from the basal viruses of the genus *Potyvirus*, which probably originated in Western Europe or north-west Africa (Gibbs and Ohshima 2010). As mentioned above, the potyvirus closest to PVY is PSMV, and judging from patristic distances in a protein sequence ML tree of known PVY lineage viruses (Fribourg et al. 2019) PVY and PSMV may have diverged around 8,000 YBP (i.e. four times the TMRCA of PVY).

In summary, our analyses of the PVY sequences provide evidence that:

1. Phylogeny:
  a. The phylogeny of complete non-recombinant PVY ORF sequences has a basal divergence that produces an N phylogroup lineage and a sister lineage that has a basal branch to the Chile 3 isolate from the pepper (*C. baccatum*) and a major lineage to the C and O phylogroups;
  b. The N phylogroup is predominantly and indigenously Andean, and only isolates from its N3 cluster have been found in plants growing outside South America (see also N3 recombinants below);
  c. O phylogroup isolates were mostly from plants growing outside South America. The few from South America are all phylogenetically distal to isolates from elsewhere, so they may be ‘remigrants’, rather than being related to a South American PVY population only via indigenous intermediate infections.
  d. The C phylogroup lineage is a monophyletic branch of the O phylogroup. No C phylogroup isolates are from South America;
  e. N and O phylogroup populations were mostly isolated from potatoes, and their ‘temporal structure’ is indistinguishable, whereas the C phylogroup isolates came from a wide range of solanaceous plants, rarely potatoes, and have evolved at least 2.5 times faster than N and O populations;
  f. Half the sequenced genomes (460—Jan 2018) are NxO recombinants, which form two phylogroups (R1 and R2). Their N phylogroup terminal regions are most closely related to those of N3 genomes rather than to either N1 or N2 genomes, and there is no evidence from these recombinants that pairs of N, C, and O isolates co-infected plants when within South America.
  g. The site of the original N population is clearly the Andes, but that of the O population is unknown, and maybe the southern Chilean *S.t.t.* population (Isle of Chiloe), which is known to be infected with PVY of which the phylogroup status is unknown.
2. Dating:
  a. The TMRCA of the non-recombinant dated world PVY population (<January 2018) is 1860 YBP or 156 CE;
  b. The dated phylogeny of PVY corresponds well with the history of the potato crop. Only the most basal divergences of the O and N phylogroups occurred before transatlantic marine trade started in the 16th century. O phylogroup isolates probably came to Europe from a South American site, that has not been identified, in the earliest transported potatoes. N phylogroup isolates came more recently and probably from the Andes, and the NxO recombination event(s) that produced the PTNRD-causing R1 and R2 recombinants occurred in the 20th century outside South America, probably in Europe;
  c. The major radiations of PVY populations probably occurred immediately after the famine-producing epidemics of late blight (*Phytophthora infestans*) in European potato crops in the mid-19th century. Subsequent potato breeding and trade probably drove virus spread and divergence.

These conclusions allow us to draft a credible history of PVY from when it diverged from PSMV possibly around 8,000 YBP, which is soon after humankind reached South America and started domesticating potatoes. Humans first spread throughout the Americas, from Beringia, at least 13,000–14,500 YBP (Dillehay et al. 2015; Wade 2018), and probably earlier. Amerindians colonized the Altiplano region around Lake Titicaca around 9,000 YBP. They domesticated potatoes by selecting and hybridizing from the diverse local tuber-bearing *Solanum* flora (Spooner et al. 2007; Ovchinnikova et al. 2011; Hardigan et al. 2017), and potatoes became a major component of the human diet of the region. Potato starch grains have been found on ground stone tools from, at least, 3,400 YBP (Rumold and Aldenderfer 2016). The people of the Aymara and Quechua language groups, who are the present inhabitants of the Altiplano region, have been shown (Lindo et al. 2018) to be genetically related to past inhabitants of the region for 4,000, and probably 7,000 years. Furthermore, all were found to have enzymic adaptations to high altitude life and a high starch diet that are not found in present day Amerindians in coastal south Chile from whom they split around 8,750 years ago. The initial populations of *S.t.a* were confined to the Lake Titicaca region, and they did not spread until later to Chiloe region in southern Chile. Their spread may have been limited by their adaptation to short day lengths (Kloosterman et al. 2013) in the Lake Titicaca region, or by the altitudinal and climatic barriers along the length of the high Andes (Hazzi et al. 2018), although they were eaten in coastal northern Peru, and stylized in the pottery of the Moche culture of that region (Duke et al. 2018), but seem not to have been taken further north than this. This restricted range, until the European invasion, is in contrast to the spread of maize over the same time period from its site of domestication in Mesoamerica, especially Mexico, to establish a secondary center of maize diversity in central South America (Kistler et al. 2018; Zeder 2018).

Potatoes, and the freeze dried ‘chuño’ made from them, helped power the many empires and cultures of the High Andes including the final Amerindian one, the Inca Empire, which lasted from 1438 to 1532 CE. Potato was fundamental to the empire's food security. It built and expanded terraces and irrigation systems, built roads that connected the empire’s different provinces and greatly facilitated trade, and fueled the empire’s increasing population (Hawkes 1978; Brown and Henfling 2014). This expansion of potato cropping, would have enabled widespread dissemination of PVY within the valleys of the Andes.

The first potatoes to be taken to Europe from South America arrived, most likely at the Canary Islands in the 1560s, and thence to other parts of Europe in the second half of the 16th century, and later to other continents (Salaman and Hawkes 1949; Salaman 1954; Hawkes 1990; Brown 1993; Hawkes and Fransisco-Ortega 1993; Brown and Henfling 2014). The initial shipments from South America were small, and there has been much lively discussion about their source. Salaman and Hawkes (1949) reasoned that potatoes would have only survived a trip direct from the Andes to the Caribbean and then to Europe, but would not have survived the longer trip from southern Chile. However genetic studies have shown that the early potatoes came from both regions (Glenndinning 1983; Ames and Spooner 2008; Rios, Rodriguez, and Spooner 2007). The culture of these hybrids then spread throughout Europe (e.g. O’Riordan 2001), where they were, at first, an oddity, but eventually they were adopted as a major food crop about 100 years later.

‘Potato degeneration’, summarized by Salaman (1925), was caused by a complex of virus diseases that was recognized in the early days of the European crop especially when potatoes were propagated by tuber from year to year rather than being grown from seed. One form of degeneration called ‘The Curl’ in the UK was clearly caused by virus infection and first described in the 1770s. The dominant cause was probably potato leaf roll virus, but one or more mosaic-causing viruses also fit some of the contemporary descriptions (Salaman 1925, 1954), and may have been the earliest recorded sightings of PVY. Selection of new cultivars from naturally formed potato seedlings, and only much later through controlled crossing, resulted in a plethora of cultivars. The earliest potato breeding programs of any size began in 1810, but the practice did not become widespread until the second half of the 19th century. It was stimulated by the disastrous epidemics of potato blight disease caused by the oomycete *P.* *infestans* from Mexico (Gossa et al. 2014; Fry et al. 2015). This appeared in the middle of the 19th century, ruined the crop, and eliminated almost all cultivars as they were so inbred and susceptible. Breeding among blight survivors, together with new introductions from South America, led to many new cultivars being grown by early 20th century (Glenndinning 1983), and distribution of these may have initiated the major radiation of the O phylogroup isolates dated as 1868 CE (Fig. 4). Thus although the initial divergence of the O lineage was well before the Inca era, most major divergences occurred after the potato crop was established in Europe. Likewise although the N phylogroup lineage first diverged in the early 16th century, most of its radiation occurred after 1899 CE (Fig. 4), and potatoes with tuber necrotic ringspot disease, which indicates the presence of R2 phylogroup recombinants (Gibbs et al. 2017) with N3 genomic termini, were not found until the early 1980s in Europe (Beczner et al. 1984; Le Romancer, Kerlan, and Nedellec 1994; Boonham et al. 2002; Lorenzen et al. 2006).

Two recent papers on potato virus S (PVS), a carlavirus, reported phylogenies with the same topology as that of PVY, namely a South American origin and a recent major European divergence, which was dated 1837 CE by LSD analysis of 35 ORF sequences (Santillan et al. 2018) and 1859 CE by BEAST analysis of 103 CP sequences (Duan et al. 2018). Thus the date for the divergence of PVS and PVY in the European potato crop is, in essence, the same, and is around or soon after the Great Potato Famine. Why the LSD dating method worked for PVS, but not PVY, is not known but may be because the host range of PVS is much more limited than that of PVY (Brunt et al. 1996), and therefore, in the wild, they probably differ in their ability to infect, and gain greater genetic diversity in, novel hosts.

There is still much to learn about the evolution of PVY. More will undoubtably be learned from the genomic sequences of more isolates from the Andean center of potato domestication, also the Chiloe region, especially ones from potato land races, wild potato species and other solanaceous crops. Likewise, sequencing of PVY isolates from the islands visited by the early trans-Atlantic traders, and the remnant stocks of early European potatoes, such as the ‘fingerling’ types, might also provide further intriguing information.

## Supplementary Material

vez037_Supplementary_Data
