# Supplementary material for: Potato virus Y; the Andean connection
Source: Virus Evol. 2019 Sep 23;5(2):vez037. doi: 10.1093/ve/vez037 (PMC6755682; doi:10.1093/ve/vez037)
Supplement: vez037_Supplementary_Data [file vez037_supplementary_data.zip › Fig S1.docx]

Fig S1

‘Residuals’ graph from a TempEst analysis of the 190 sequence dataset showing the large ‘residuals’ (right of the dashed vertical line) generated by C phylogroup sequences. These and others were removed to generate the 162 sequence dataset used for dating analysis.
