# Supplementary material for: Potato virus Y; the Andean connection
Source: Virus Evol. 2019 Sep 23;5(2):vez037. doi: 10.1093/ve/vez037 (PMC6755682; doi:10.1093/ve/vez037)
Supplement: vez037_Supplementary_Data [file vez037_supplementary_data.zip › Supplementary_Data_2.docx]

**Supplementary data:**

Database (CSV format) of:

Acc Code: Accession Codes of the sequences used for the analyses;

phylo gp: phylogroup to which the sequence belongs;

date: the ‘collection date’, CE;

prov: country of provenance;

host: *Capsicum annuum; C. baccatum; Kalmeris indica; Nicotiana tabacum; Physalis peruvianum; Solanum americanum; S. lycopersicum; S. sisymbriifolium, S. tuberosum. andigena, S. tuberosum. tuberosum* and *Vitis vinifera*;

190 set: sequences in the 190 sequence dataset;

162 set: sequences in the 162 sequence datasets.

Latitude of collection site

Longitude of collection site

Fig 1

‘Residuals’ graph from a TempEst analysis of the 190 sequence dataset showing the large ‘residuals’ (right of the dashed vertical line) generated by C phylogroup sequences. These and others were removed to generate the 162 sequence dataset used for dating analysis.
